# Predictors of depression outcomes among university students following brief smartphone-based interventions

**DOI:** 10.1038/s44184-026-00208-3

**Published:** 2026-04-17

**Authors:** Xuanchen Liu, WuYi Zheng, Leonard Hoon, Sunil Gupta, Svetha Venkatesh, Helen Christensen, Jill Newby, Alexis E. Whitton

**Affiliations:** 1 Black Dog Institute, UNSW Sydney, Sydney, NSW Australia; 2 Discipline of Psychiatry and Mental Health, UNSW Sydney, Sydney, NSW Australia; 3 Applied Artificial Intelligence Initiative, Deakin University, Melbourne, VIC Australia; 4 School of Psychology, UNSW Sydney, Sydney, NSW Australia; 5 Centre for Contextual Psychiatry, Department of Neuroscience, KU Leuven, Leuven, Belgium; 6 Centre for the Health Economy, Macquarie University, Sydney, NSW Australia; 7 Centre for Digital Transformation of Health, The University of Melbourne, Melbourne, VIC Australia; 8 Division of Arts and Sciences and Centre for Global Health Equity, New York University Shanghai, Shanghai, PR China; 9 Westmead Institute for Medical Research, University of Sydney, Sydney, NSW Australia

**Keywords:** Diseases, Health care, Medical research, Psychology, Psychology

## Abstract

Smartphone-delivered interventions offer a scalable solution for university students experiencing depression, yet outcomes remain inconsistent. This study examined predictors of depression remission and response in the AI-enhanced Vibe Up adaptive trial of brief smartphone-based interventions in 1282 Australian university students (mean age 23.52 years; 78.39% women) with elevated distress (Kessler-10 ≥ 20). After a two-week monitoring period, participants were randomised to two-week self-guided smartphone-based interventions targeting sleep hygiene, mindfulness, physical activity, or an ecological momentary assessment control intervention. Predictors of depression remission (DASS-21 in normal range) and response (≥50% reduction) were examined using hierarchical logistic regression. At post-intervention, 40.87% achieved remission and 29.88% showed response. Baseline depression severity, quality of life, general practitioner visits, and pre-intervention credibility predicted remission and/or response across all intervention arms. Higher baseline anxiety specifically predicted poorer remission in the sleep hygiene arm. Individual factors modestly predicted outcomes. Trajectory-based predictors may be needed to improve outcome prediction.

## Introduction

Depression is a prevalent mental health condition and the second highest cause of disability globally^1–4^. University students are a particularly high-risk group for depression, with around one third worldwide reporting symptoms of depression^5^. Among the broader constellation of psychological distress, which includes depression, anxiety and stress, depression is one of the most impairing components^6,7^. It is associated not only with the development of serious mental disorders, but also with a range of adverse outcomes, including academic underperformance and university dropout^8,9^, increased substance misuse^10^, and a heightened risk of suicidal ideation and behaviour^11^. However, many students still do not receive the support they need^12^.

Although psychotherapies, such as Cognitive Behavioural Therapy (CBT), and pharmacotherapy are effective treatments for depression^13^, their accessibility is often constrained by long wait times, high costs and a shortage of skilled clinicians^14–16^. To overcome these limitations, there has been increasing interest in digital interventions that deliver lifestyle or psychological interventions (such as sleep hygiene, mindfulness, and physical activity) through accessible and convenient digital platforms^17–19^. Nevertheless, their effectiveness varies among individuals, underscoring the need to better understand how to personalise treatments^20,21^. However, research identifying individual-level factors that can inform the personalisation of digital interventions remains scarce.

Identifying predictors of intervention remission and response to digital interventions is crucial, as it helps distinguish subgroups of patients who respond differently to interventions, thereby enabling more precise and personalised care^22^. Predictors can be divided into two categories: prognostic and prescriptive^22,23^. Prognostic predictors are characteristics that indicate the likelihood of better or worse outcomes, regardless of the type of treatment received^24,25^. These factors are useful for identifying individual risk, informing triage and care stratification, and guiding allocation of resource-intensive interventions or the tailoring of treatment intensity to individuals or subgroups with similar prognostic profiles^26^. By contrast, prescriptive predictors, also referred to as moderators, are characteristics that identify individuals who are likely to respond better or worse to specific treatments. These factors help guide treatment selection by predicting differential responses to particular interventions^27,28^.

Despite growing interest in prognostic predictors of treatment outcomes, findings remain inconclusive. Baseline depression severity is one of the most commonly examined clinical predictors, but findings regarding its association with treatment outcomes are mixed. Some studies associate higher baseline severity with higher treatment response but lower remission rates, while others find associations with lower response or no significant association^29–32^. In digital intervention settings, higher baseline severity has been linked to greater symptom improvement. Common to these studies is the use of dimensional symptom change scores as the outcome measure of interest^33–35^, as opposed to the binarised clinical depression remission or response outcomes that are often used in other intervention contexts (e.g., pharmacological trials)^36,37^.

Demographic and identity-related variables, such as sex, socioeconomic status (SES), sexual orientation, and culturally and linguistically diverse (CALD) background, have also been examined as potential prognostic predictors of depression treatment outcomes^38–42^, but again, findings have been mixed. For instance, some studies suggest that women may experience more favourable outcomes than men, while others find no significant predictive value of sex or gender^42–44^. Psychosocial variables such as perceived social support^22,45^ and quality of life^46^, and treatment-related variables such as treatment credibility and expectancy^47,48^ have also been identified as variables associated with better treatment outcomes. However, evidence of the predictive utility of these variables in the digital interventions setting remains limited and requires further study.

In contrast to the research on prognostic predictors, there is relatively limited evidence examining prescriptive predictors. Of the limited studies available, findings suggest that baseline depression as well as baseline anxiety severity may influence responsiveness to specific forms of treatment. For instance, researchers found that baseline depression/anxiety severity and chronicity moderated response to Psychodynamic Supportive Psychotherapy (SPSP) and CBT^49^. Specifically, SPSP was found to be more effective for individuals with mild-to-moderate depression and low anxiety, or for those with chronic episodes, whereas CBT was found to be more effective for individuals with acute severe depression. Ethnic background has also been examined as a potential prescriptive predictor: one study reported that combined CBT and medication was more effective than medication alone for White adolescents, but not for their non-White counterparts^50^. However, other research found no such effects^51^. Some studies have found that variables such as being married, unemployed, or having experienced recent life events may also be associated with better outcomes from psychotherapy than from pharmacological interventions^24,52^, however, findings are not consistent across studies.

To our knowledge, only one study has specifically examined prescriptive effects of treatment outcomes in self-guided digital interventions for depression. In this study, researchers found that in university students with symptoms of depression, baseline exercise habits moderated response to a digital self-monitoring module, with greater benefits of self-monitoring observed among those who were less active at baseline^35^. However, the study did not directly compare outcomes with other therapy modules, so it remains unclear whether lower baseline exercise predicted better response to self-monitoring relative to other therapeutic techniques, or better overall prognosis more generally. The absence of robust predictors in this field limits the development of effective, personalised intervention strategies.

To address these gaps, the present study used data from the Vibe Up trial^53,54^. This was a four-arm AI-enhanced adaptive trial comparing the effectiveness of three brief digital interventions (sleep hygiene, mindfulness, and physical activity) with an ecological momentary assessment (EMA) control condition, for reducing psychological distress (symptoms of depression, anxiety or stress) among university students. Findings from the trial indicated that physical activity and mindfulness were most effective for students with severe psychological distress, and physical activity and sleep hygiene were most effective for those with mild distress. However, no significant differences between interventions were found for those with moderate distress, and high heterogeneity in symptom profiles within this group was identified as a potential explanation for these null findings^54^. To address this limitation, the present study examined outcomes at the symptom-domain level, focusing on the most prevalent symptom domain in the cohort—depression—rather than relying on a composite distress score. Specifically, we focused on identifying prognostic and prescriptive predictors of improvement in depression, using the widely applied binarised outcomes of depression treatment remission and response.

By identifying both prognostic and prescriptive predictors of remission and response, this study aimed to contribute to the evidence base to inform more personalised and effective brief digital interventions for depression in university student populations. Guided by previous research, we assessed a range of prognostic and prescriptive predictors, including demographic, clinical, psychosocial, treatment-related, and identity-related variables. We hypothesised that lower baseline depression severity, higher quality of life, higher perceived social support, higher SES, and stronger treatment expectancy would be associated with increased likelihood of remission and response. Given the lack of studies identifying prescriptive predictors of remission/response in digital mental health interventions, strong hypotheses could not be made. However, this study focused specifically on baseline depression and anxiety severity, as these are key variables commonly examined as prescriptive predictors in prior research^55,56^.

## Methods

This secondary analysis was conducted using data from the Vibe Up trial, which was approved by UNSW Sydney Human Research Ethics Committee (HC200466), and registered with the Australian New Zealand Clinical Trials Registry (ACTRN12621001223820; registered on 13-Sep-2021). Comprehensive details regarding recruitment, participants, measures, interventions, study design, and procedures (including monitoring timelines) are available in the published trial protocol^53^, and outcomes paper^54^. The trial was reported in accordance with the Consolidated Standards of Reporting Trials guidelines^54^.

### Participants

Participants were adults aged 18 or older, currently residing in Australia, enrolled at a higher education institution, and had provided electronic informed consent. All were fluent in English, owned an eligible smartphone (iPhone 6S/Android 5 or later) with an active mobile number and internet access, and scored ≥ 20 on the 10-item Kessler Psychological Distress Scale (K-10), indicative of likely mild or more serious mental distress.

### Outcome measures

Baseline demographic and screening questionnaires were administered via Qualtrics (Provo, UT), while all pre- and post-intervention self-report measures were delivered through the Vibe Up smartphone app.

Depression remission was assessed using the post-intervention depression subscale of the 21-item Depression Anxiety Stress Scales (DASS-21). To align with the original DASS-42 scoring system, raw DASS-21 scores were multiplied by two, and remission was defined as a score of 9 or lower on the adjusted scale^57^.

Depression response was measured using the pre- and post-intervention DASS-21 depression subscale, with response defined as a reduction of 50% or more in depressive symptoms from pre- to post-intervention^32^.

### Demographic predictors

Sex was measured at baseline using a self-report item asking participants to indicate their sex recorded at birth (Female, Male, or another term).

SES was measured at baseline using a visual ladder, where participants were asked to place themselves on a 10-rung ladder representing their perceived standing in the Australian society, based on income, education, and job respect^58^. A higher rung indicated higher perceived social standing.

### Clinical predictors

Baseline symptom severity was assessed using the DASS-21, which assesses depression, anxiety, and stress^59^. Scores on the depression and anxiety subscales were used to index baseline depression and anxiety severity, respectively.

Mental health service use was assessed at baseline using the frequency of general practitioner (GP) visits for mental health concerns in the past 12 weeks, as GPs in Australia often serve as the first point of contact for mental health care^60,61^. Other forms of service use, such as visits to psychologists or psychiatrists, were also assessed; however, most participants reported no or minimal use of these services, so analyses focused primarily on GP visits.

### Psychosocial predictors

Perceived social support was measured at baseline using the Multidimensional Scale of Perceived Social Support (MSPSS)^62^, a validated 12-item measure assessing support from family, friends, and significant others. Items were rated on a 7-point Likert scale ranging from Very strongly disagree (1) to Very strongly agree (7).

Quality of life was measured at baseline using the Recovering Quality of Life-10 (ReQoL-10), a scale designed to assess mental health-related quality of life, particularly in individuals experiencing common mental health difficulties^63^.

### Treatment-related predictors

Two items drawn from the Credibility and Expectancy Questionnaire (CEQ) were used to assess pre-intervention treatment credibility and expectancy^64^. Credibility was assessed by asking participants how logical they perceived the app to be for improving distress, rated on a 9-point Likert scale (1 = not at all logical, 9 = very logical). Expectancy was assessed using a separate item asking participants to estimate the degree of improvement they genuinely expected, rated on an 11-point scale from 0% to 100% in 10% increments.

### Identity-related predictors

LGBTQA+ status was measured at baseline based on participants’ self-identified sexual orientation, with individuals classified as LGBTQA+ if they selected “Gay or lesbian”, “Bisexual” or indicated “I use a different term.”

CALD background was measured at baseline and defined as speaking a language other than English at home or reporting an ancestry other than Australian, British, or Irish. This definition aligns with prior research in Australian mental health settings^65^, and enables the inclusion of second-generation migrants who may be culturally and linguistically diverse but born in Australia.

### Interventions

All interventions were brief, self-guided, and delivered via the Vibe Up smartphone app. The EMA control condition involved twice-daily mood surveys adapted from the International Positive and Negative Affect Short-Form^66^. The sleep hygiene intervention used infographics to promote healthy sleep habits, covering topics such as sleep environment, routine, and the role of activity and diet^67^. The mindfulness intervention included a short video and five daily guided audio meditations (<5 min) promoting present-moment awareness, non-judgement, and self-compassion. The physical activity intervention featured an infographic on exercise benefits and how to increase activity levels, goal setting (e.g., step count), and a 7-min high intensity interval training video^68^.

### Randomisation and Allocation

Allocation was determined either by sequential assignment based on the order of baseline assessment completion (for participants in the first mini-trial), or by a contextual multi-arm bandit AI algorithm (for participants in mini-trials 2–12)^69^. Full details of the adaptive randomisation process have been reported elsewhere^53,54^.

### Statistical analysis

All analyses were conducted in R 4.4.2. All *p* values were from 2-sided tests, and results were deemed statistically significant at *p* < 0.05.

Among the 1282 participants, missing data were observed for depression remission (*n* = 156, 12.17%), depression response (*n* = 158, 12.32%), distress remission (*n* = 156, 12.17%), distress response (*n* = 156, 12.17%), ReQoL-10 (*n* = 101, 7.88%), and CEQ (*n* = 60, 4.68%). Missingness was associated with demographic characteristics (age, sex, and CALD background), supporting the assumption that data were Missing at Random (MAR; see Tables S1–S2). A small proportion of participants selected “don’t know” or “prefer not to say” for LGBTQA+ status (*n* = 75, 5.85%), which was treated as missing. However, no substantial bias was detected in missingness patterns (see Table S3). Missing data were addressed using multiple imputation by chained equations (MICE), generating 50 imputed datasets^70^. The imputation model included all analysis variables and auxiliary predictors of missingness. Estimates were pooled using Rubin’s rules^71^.

Given the theory-informed nature of this study, predictors were entered into hierarchical logistic regression models in conceptually defined blocks to identify prognostic predictors of remission and response. In all models, treatment condition was included as a control variable to account for intervention effects, treated as a categorical variable, with EMA as the reference group. Mini-trial number (1–12) was also treated as a control variable to account for the effects of the adaptive treatment allocation. Continuous predictors were entered in their original scale.

In step 1, demographic variables (e.g., sex and SES) were entered. Step 2 added clinical and psychosocial variables (e.g., baseline depression severity, perceived social support, quality of life and GP visit frequency). Step 3 further included treatment-related (e.g., treatment credibility and expectancy) and identity-related (e.g., LGBTQA+ status and CALD background) variables.

Consistent with the stratified design of the parent trial^54^, exploratory subgroup analyses were conducted by estimating separate hierarchical logistic regression models within each baseline severity group to assess potential variation in prognostic predictor patterns.

Prescriptive predictors were examined exploratorily by including interaction terms between treatment condition and baseline factors in step 3 of the hierarchical models. Each candidate variable was entered separately as an interaction term with treatment condition. A comprehensive list of all prescriptive predictors examined is presented in Table S4. For significant or near-significant interactions, exploratory pairwise contrasts between treatment conditions were assessed using estimated marginal means. Because interaction effects with continuous moderators cannot be interpreted as a single effect size, treatment contrasts were evaluated at representative values of the moderator (mean and ± 1 standard deviation). Odds ratios (ORs) and 95% confidence intervals (CIs) were derived on the logit scale. These contrasts were conducted without adjustment for multiple comparisons and were considered as exploratory.

Multicollinearity was assessed using variance inflation factors (VIF < 5). Model fit was evaluated using likelihood ratio tests and both Cox-Snell and Nagelkerke pseudo-*R*² statistics.

## Results

### Baseline characteristics of participants

The sample comprised *N* = 1282 university students with mild to severe psychological distress, with a mean age of 23.52 years (*SD* = 5.21). Most participants were female (*n* = 1005; 78.39%). See Table 1 for baseline characteristics by intervention arm.

**Table 1:** Sample demographics

	Ecological Momentary Assessment (*n* = 93)	Sleep Hygiene (*n* = 431)	Mindfulness (*n* = 453)	Physical Activity (*n* = 305)	Overall (*N* = 1282)
Age, mean (*SD*)	22.89 (4.12)	23.96 (5.63)	23.28 (4.95)	23.46 (5.22)	23.52 (5.21)
SES, mean (*SD*)	5.63 (1.89)	5.97 (1.78)	5.73 (1.82)	5.82 (1.85)	5.82 (1.82)
Baseline Depression Severity, mean (*SD*)	17.81 (11.01)	15.27 (8.31)	19.44 (8.94)	15.53 (8.68)	16.99 (9.04)
GP Visit Frequency, mean (*SD*)	1.11 (1.77)	0.60 (1.15)	0.87 (1.48)	0.64 (1.08)	0.74 (1.29)
Perceived Social Support, mean (*SD*)	4.78 (1.15)	4.86 (1.10)	4.75 (1.11)	4.92 (1.04)	4.83 (1.09)
Quality of Life, mean (*SD*)	24.37 (7.00)	25.21 (5.72)	23.00 (5.71)	24.84 (5.74)	24.27 (5.88)
Treatment Credibility, mean (*SD*)	5.50 (1.64)	6.14 (1.61)	5.96 (1.62)	6.08 (1.56)	6.02 (1.61)
Treatment Expectancy, mean (*SD*)	34.22% (19.94%)	38.20% (19.05%)	39.05% (19.05%)	40.24% (19.57%)	38.69% (19.28%)
Sex, *n* (%)					
Female	80 (86.02)	325 (75.41)	366 (80.79)	234 (76.72)	1005 (78.39)
Male	13 (13.98)	105 (24.36)	87 (19.21)	70 (22.95)	275 (21.45)
Did not disclose	0 (0.00)	1 (0.23)	0 (0.0)	1 (0.33)	2 (0.16)
LGBTQA+ Status, *n* (%)					
Yes	41 (44.09)	140 (32.48)	177 (39.07)	109 (35.74)	467 (36.43)
No	41 (44.09)	269 (62.41)	245 (54.08)	185 (60.66)	740 (57.72)
Did not disclose	11 (11.83)	22 (5.10)	31 (6.84)	11 (3.61)	75 (5.85)
CALD Status, *n* (%)					
Yes	42 (45.16)	169 (39.21)	172 (37.97)	123 (40.33)	506 (39.47)
No	51 (54.84)	262 (60.79)	281 (62.03)	182 (59.67)	776 (60.53)

A comprehensive list of sample characteristics is reported in the main outcomes paper^54^.

### Rates of remission and response

At post-intervention, 524 (40.87%) participants met criteria for remission, and 389 (29.88%) met criteria for response.

Remission rates showed some variation across groups, with 34 (36.30%) participants in EMA, 193 (44.88%) in sleep hygiene, 151 (33.42%) in mindfulness, and 145 (47.64%) in physical activity meeting criteria for remission. However, these differences were not statistically significant (all *ps* > 0.063).

Response rates varied significantly across treatment groups. The lowest rate was observed in the EMA group (*n* = 16, 17.31%), while rates were higher in the sleep hygiene (*n* = 126, 29.35%), mindfulness (*n* = 133, 29.33%), and physical activity (*n* = 108, 35.38%) groups. Compared to the EMA group, participants in all three active treatment groups had significantly higher odds of response: sleep hygiene (OR = 1.99, 95%CI [1.08, 3.65], *p* = .027), mindfulness (OR = 1.99, 95%CI [1.08, 3.65], *p* = .028), and physical activity (OR = 2.62, 95%CI [1.42, 4.83], *p* = .002).

### Prognostic predictors of remission

Three hierarchical logistic regression models were run to identify prognostic predictors of remission in the full imputed sample (Table 2).

**Table 2:** Prognostic predictors of depression remission: hierarchical logistic regression (pooled estimates, *N* = 1282)

	B (SE)	OR [95% CI]	*p*-value
Model 1			
Sex (ref = female)	−0.27 (0.16)	0.76 [0.56, 1.04]	0.084
Socioeconomic Status	0.15 (0.04)	1.16 [1.08, 1.24]	<0.001
Model 2			
Sex (ref = female)	−0.12 (0.18)	0.89 [0.62, 1.27]	0.506
Socioeconomic Status	0.01 (0.04)	1.01 [0.93, 1.10]	0.728
Baseline Depression Severity	−0.12 (0.01)	0.89 [0.87, 0.91]	<0.001
Perceived Social Support	0.10 (0.07)	1.10 [0.96, 1.27]	0.182
Recovering Quality of Life	0.06 (0.02)	1.06 [1.02, 1.09]	0.002
GP Visit Frequency	−0.15 (0.07)	0.86 [0.75, 0.99]	0.034
Model 3			
Sex (ref = female)	−0.19 (0.19)	0.83 [0.57, 1.19]	0.312
Socioeconomic Status	0.00 (0.04)	1.00 [0.92, 1.09]	0.941
Baseline Depression Severity	−0.12 (0.01)	0.89 [0.87, 0.91]	<0.001
Perceived Social Support	0.11 (0.07)	1.12 [0.97, 1.29]	0.125
Recovering Quality of Life	0.05 (0.02)	1.05 [1.02, 1.09]	0.004
GP Visit Frequency	−0.14 (0.07)	0.87 [0.76, 1.00]	0.052
Pre-intervention Credibility	0.04 (0.05)	1.04 [0.94, 1.14]	0.456
Pre-intervention Expectancy	0.01 (0.00)	1.01 [1.00, 1.01]	0.167
LGBTQA+ Status	−0.29 (0.16)	0.75 [0.55, 1.02]	0.064
CALD Background	0.16 (0.15)	1.18 [0.88, 1.58]	0.269

Model 1: Mean *R*² = 0.04 (Cox-Snell), 0.05 (Nagelkerke), *χ*²(6) = 48.06, *p* < 0.001. Model 2: Mean *R*² = 0.25 (Cox-Snell), 0.33 (Nagelkerke), *χ*²(10) = 365.82, *p* < 0.001. Model 3: Mean *R*² = 0.25 (Cox-Snell), 0.34 (Nagelkerke), *χ*²(13) = 373.07, *p* < 0.001. Treatment condition and mini-trial number were included in the model as control variables, but are not interpreted here. The intercept is omitted. B = unstandardised logistic regression coefficient (log odds); *SE* = standard error, *OR* = odds ratio, *CI* = confidence interval. OR > 1 indicates higher odds of remission (vs. no remission).

Model 1 focused on demographic variables (sex and SES) as key prognostic factors of interest. The overall model was significant, *χ*²(6) = 48.06, *p* < 0.001, Nagelkerke *R*² = 0.05. Results showed that higher SES (range = 0–10; OR = 1.16, 95% CI [1.08, 1.24], *p* < 0.001) significantly predicted higher odds of remission.

Model 2 included the same variables as Model 1, but added clinical and psychosocial variables as additional prognostic factors of interest. An improvement in model fit was observed, *χ*²(10) = 365.82, *p* < 0.001, with Nagelkerke *R*² increasing to 0.33. Results showed that higher baseline depression severity (range = 0–42; OR = 0.89, 95% CI [0.87, 0.91], *p* < 0.001) and more mental health-related GP visits (range = 0–17; OR = 0.86, 95% CI [0.75, 0.99], *p* = 0.034) were significantly associated with lower odds of remission, whereas higher quality of life was associated with higher odds of remission (range = 3–41; OR = 1.06, 95% CI [1.01, 1.09], *p* = 0.034).

Model 3 included the same variables as Models 1 and 2, but also introduced treatment-related credibility and expectancy beliefs, and sexual and cultural identity-related variables. The final model remained significant, *χ*²(13) = 373.07, *p* < 0.001, with Nagelkerke *R*² increasing slightly from 0.33 to 0.34. Results showed that lower baseline depression severity (range = 0–42; OR = 0.89, 95% CI [0.87, 0.91], *p* < 0.001) and higher quality of life (range = 3–41; OR = 1.05, 95% CI [1.02, 1.09], *p* = 0.004) remained significantly associated with higher odds of remission.

### Prognostic predictors of treatment remission by baseline symptom severity group

Separate models were also estimated for participants with different baseline distress severity (see Tables S5a–S5c).

Results showed that in the mild distress group, higher baseline depression severity (range = 0–42; OR = 0.88, 95% CI [0.84, 0.92], *p* < 0.001) was associated with lower odds of remission. In the moderate group, higher baseline depression severity (OR = 0.88, 95% CI [0.83, 0.92], *p* < 0.001) and more frequent mental health-related GP visits (range = 0–17; OR = 0.75, 95% CI [0.59, 0.96], *p* = 0.024) were linked to lower odds of remission. In the severe group, higher baseline depression severity (range = 0–42; OR = 0.89, 95% CI [0.84, 0.95], *p* < 0.001) was associated with lower odds of remission, and higher perceived social support (range = 1–7; OR = 1.42, 95% CI [1.02, 1.97], *p* = 0.039) was associated with higher odds of remission.

### Prognostic predictors of response

Hierarchical logistic regression models were also used to examine prognostic predictors of treatment response (Table 3).

**Table 3:** Prognostic predictors of treatment response: hierarchical logistic regression (pooled estimates, *N* = 1282)

	B (SE)	OR [95% CI]	*p*-value
Model 1			
Sex (ref = female)	0.01 (0.16)	1.01 [0.73, 1.40]	0.952
Socioeconomic Status	0.05 (0.04)	1.05 [0.98, 1.13]	0.158
Model 2			
Sex (ref = female)	0.08 (0.17)	1.08 [0.77, 1.52]	0.639
Socioeconomic Status	0.01 (0.04)	1.01 [0.93, 1.09]	0.894
Baseline Depression Severity	−0.04 (0.01)	0.96 [0.94, 0.98]	<0.001
Perceived Social Support	0.09 (0.07)	1.10 [0.96, 1.26]	0.190
Recovering Quality of Life	−0.01 (0.02)	0.99 [0.96, 1.02]	0.572
GP Visit Frequency	−0.13 (0.07)	0.87 [0.77, 1.00]	0.042
Model 3			
Sex (ref = female)	0.06 (0.17)	1.06 [0.75, 1.50]	0.729
Socioeconomic Status	−0.00 (0.04)	1.00 [0.92, 1.08]	0.944
Baseline Depression Severity	−0.04 (0.01)	0.96 [0.94, 0.98]	<0.001
Perceived Social Support	0.09 (0.07)	1.09 [0.95, 1.25]	0.209
Recovering Quality of Life	−0.01 (0.02)	0.99 [0.96, 1.02]	0.472
GP Visit Frequency	−0.14 (0.07)	0.87 [0.77, 1.00]	0.044
Pre-intervention Credibility	0.10 (0.05)	1.10 [1.01, 1.21]	0.039
Pre-intervention Expectancy	0.00 (0.00)	1.00 [0.99, 1.01]	0.785
LGBTQA+ Status	−0.01 (0.14)	0.99 [0.74, 1.31]	0.939
CALD Background	−0.00 (0.14)	1.00 [0.76, 1.31]	0.990

Model 1: Mean *R*² = 0.02 (Cox-Snell), 0.02 (Nagelkerke), *χ*²(6) = 19.58, *p* = 0.005. Model 2: Mean *R*² = 0.05 (Cox-Snell), 0.06 (Nagelkerke), *χ*²(10) = 59.38, *p* < 0.001. Model 3: Mean *R*² = 0.05 (Cox-Snell), 0.07 (Nagelkerke), *χ*²(13) = 61.90, *p* < 0.001. Treatment condition and mini-trial number were included in the model as control variables, but are not interpreted here. The intercept is omitted. B = unstandardised logistic regression coefficient (log odds); *SE* = standard error, *OR* = odds ratio, *CI* = confidence interval. OR > 1 indicates higher odds of response (vs. no response).

Model 1 included control and demographic variables. The results showed that, though the model was significant, *χ*²(6) = 19.58, *p* = 0.005, Nagelkerke *R*² = 0.02, no demographic variables were individually significant predictors of response.

Model 2 added baseline clinical and psychosocial variables. Compared to Model 1, Model 2 provided better fit, *χ*²(10) = 59.38, *p* < 0.001, Nagelkerke *R*² = 0.06. Results showed that higher baseline depression severity (range = 0–42; OR = 0.96, 95% CI [0.94, 0.98], *p* < 0.001) and more frequent GP visits (range = 0–17; OR = 0.87, 95% CI [0.77, 1.00], *p* = 0.042) were significantly associated with lower odds of response.

Model 3 added treatment beliefs and identity-related variables. The model remained significant, *χ*²(13) = 61.70, *p* < 0.001, with Nagelkerke R² increasing slightly from 0.06 to 0.07. Results showed that higher baseline depression severity (range = 0–42; OR = 0.96, 95% CI [0.94, 0.98], *p* < 0.001) and more frequent GP visits (range = 0–17; OR = 0.87, 95% CI [0.77, 1.00], *p* = 0.044) continued to be significantly associated with lower odds of response. Higher pre-intervention treatment credibility was significantly associated with higher odds of response (range = 1–9; OR = 1.10, 95% CI [1.01, 1.21], *p* = 0.039).

### Prognostic predictors of treatment response by baseline symptom severity group

Separate models were run for mild, moderate, and severe baseline distress subgroups (see Tables S6a–S6c).

Results showed that in the mild distress group, higher baseline depression severity (range = 0–42; OR = 0.95, 95% CI [0.91, 0.99], *p* = 0.022) was associated with lower odds of response. In the moderate group, higher baseline depression severity (range = 0–42; OR = 0.94, 95% CI [0.90, 0.99], *p* = 0.025) and more frequent GP visits (range = 0–17; OR = 0.72, 95% CI [0.55, 0.94], *p* = 0.016) were both significantly associated with lower odds of response. In the severe group, higher baseline depression severity (range = 0–42; OR = 0.94, 95% CI [0.90, 0.99], *p* = 0.013) was significantly associated with lower odds of response.

### Prescriptive predictors of remission

To evaluate prescriptive predictors of depression remission, we tested whether the effect of treatment on remission was moderated by baseline anxiety severity. Treatment condition was coded as a four-level factor (EMA, sleep hygiene, mindfulness, physical activity), with treatment contrasts applied and EMA set as the reference group (Table 4; see the full table in Table S7). In Model 1, results indicated that no interaction terms were statistically significant. However, in Model 2, a significant interaction was observed between baseline anxiety severity (range = 0–38) and the sleep hygiene intervention (OR = 0.90, 95% CI [0.83, 0.97], *p* = 0.009), indicating reduced remission likelihood for participants with higher anxiety receiving the sleep hygiene intervention compared to those with similar anxiety levels receiving EMA. This interaction remained significant in the full model (Model 3; OR = 0.90, 95% CI [0.83, 0.97], *p* = 0.009), after adjusting for other variables. No other treatment-by-anxiety interactions reached statistical significance.

**Table 4:** Interaction effects between treatment condition and baseline anxiety severity predicting remission

Interaction Term	B (SE)	OR [95% CI]	*p*-value
Sleep Hygiene × Anxiety	−0.11 (0.04)	0.90 [0.83, 0.97]	0.009
Mindfulness × Anxiety	−0.03 (0.04)	0.97 [0.90, 1.05]	0.483
Physical Activity × Anxiety	−0.01 (0.04)	0.99 [0.92, 1.07]	0.796

This is the Model 3 pooled results. B = unstandardised logistic regression coefficient (log odds); *SE* = standard error, *OR* = odds ratio, *CI* = confidence interval. All treatment effects are reported relative to the EMA condition.

To facilitate interpretation of this interaction, exploratory pairwise contrasts between treatment conditions were estimated at different levels of baseline anxiety (mean ± 1 SD). At low levels of anxiety (score = 5.09), no significant differences between treatment conditions were observed. At moderate levels of anxiety (score = 12.67), sleep hygiene was associated with lower odds of remission relative to physical activity (OR = 0.64, 95% CI [0.43, 0.95], *p* = .027). At higher levels of anxiety (score = 20.24), sleep hygiene was associated with lower odds of remission relative to physical activity (OR = 0.31, 95% CI [0.16, 0.62], *p* = 0.001), and mindfulness (OR = 0.44, 95% CI [0.24, 0.83], *p* = 0.011). EMA was associated with higher odds of remission relative to sleep hygiene (OR = 2.79, 95% CI [1.10, 7.10], *p* = 0.031). Detailed results are presented in Table S8.

**Fig. 1:**
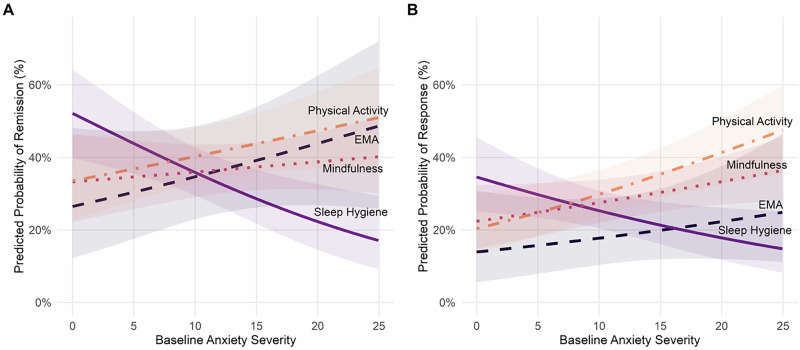
Pooled predicted probability of remission and response by baseline anxiety severity and treatment condition. Panel **A** shows predicted probabilities of remission (%) against baseline anxiety severity across interventions, and Panel **B** shows predicted probabilities of response (%) against baseline anxiety severity across interventions. Lines represent different treatment conditions (physical activity, EMA, mindfulness, and sleep hygiene), and shaded areas indicate 95% confidence intervals. Predicted values are based on pooled logistic regression across 50 imputed datasets. Each model includes treatment × anxiety interaction, adjusted for mini-trial number, sex, SES, baseline depression severity, mental health-related quality of life, perceived social support, GP visit frequency, treatment-related variables and identity-related variables.

Figure 1A illustrates the predicted probability of remission across levels of baseline anxiety severity for each intervention. As shown, the sleep hygiene intervention exhibited a steeper decline in remission probability with increasing anxiety severity, supporting a significant moderation effect. Other interventions showed relatively stable or non-significant trends.

### Prescriptive predictors of response

To explore whether treatment effects on response also varied by baseline anxiety severity, interaction terms between treatment condition and baseline anxiety severity were tested across three models (Table 5, see the full table in Table S9). In Model 1, results showed that no interaction terms were statistically significant. However, in Model 2, a marginal significant interaction was found between the sleep hygiene intervention and baseline anxiety (OR = 0.93, 95% CI [0.86, 1.00], *p* = 0.063). This interaction remained marginal significant in the full model (Model 3; OR = 0.92, 95% CI [0.86, 1.00], *p* = 0.061), after adjusting for other variables. Results showed a similar pattern to those observed in the remission analyse, with the exception of contrasts involving the EMA condition (Fig. 1B).

**Table 5:** Interaction effects between treatment condition and baseline anxiety severity predicting response

Interaction term	B (SE)	OR [95% CI]	*p*-value
Sleep Hygiene × Anxiety	−0.07 (0.04)	0.93 [0.86, 1.00]	0.061
Mindfulness × Anxiety	−0.00 (0.04)	1.00 [0.93, 1.07]	0.975
Physical Activity × Anxiety	0.02 (0.04)	1.02 [0.95, 1.10]	0.551

This is the Model 3 pooled results. B = unstandardised logistic regression coefficient (log odds); *SE* = standard error, *OR* = odds ratio, *CI* = confidence interval. All treatment effects are reported relative to the EMA condition.

Exploratory pairwise contrasts between treatment conditions were also estimated at different levels of baseline anxiety (mean ± 1 SD). At low levels of anxiety (score = 5.09), no significant differences between treatment conditions were observed. At moderate levels of anxiety (score = 12.67), EMA was associated with lower odds of response relative to physical activity (OR = 0.48, 95% CI [0.25, 0.91], *p* = 0.025). Sleep hygiene was associated with lower odds of response relative to physical activity (OR = 0.62, 95% CI [0.43, 0.89], *p* = 0.010). At higher levels of anxiety (score = 20.24), sleep hygiene was associated with lower odds of remission relative to physical activity (OR = 0.30, 95% CI [0.16, 0.55], *p* < 0.001), and mindfulness (OR = 0.43, 95% CI [0.24, 0.76], *p* = 0.004). EMA was associated with lower odds of response relative to physical actibity (OR = 0.41, 95% CI [0.18, 0.93], *p* = 0.032). Detailed results are presented in Table S10.

We also tested interactions between treatment condition and a range of baseline variables, including age, SES, employment, baseline distress severity, baseline depression severity, mental health services use, muti-diagnosis, readiness to change, helplessness, LGBTQA+ status, and CALD status. However, none of these interactions was statistically significant predictors of treatment remission or response in the hierarchical models. Full results for all tested interaction terms are reported in Tables S11–S12.

### Sensitivity analyses

Given the initial criteria for inclusion in the Vibe Up trial were elevated distress scores on the K-10 (rather than elevated depression scores specifically), some participants (*n* = 277, 21.61%) had depression scores that were already within the range used to define remission at baseline (DASS-21 depression score in the normal range). Therefore, we conducted a sensitivity analysis restricted to those with elevated baseline depression (DASS-21 depression score above the normal range) to examine prognostic and prescriptive factors of remission. The findings we observed were consistent with those derived from the full sample (See Tables S13–S14), indicating that our results were not unduly influenced by the proportion of participants presenting with minimal depression symptoms at baseline.

To examine alignment with the Vibe Up trial’s broader focus on psychological distress^54^, we conducted supplementary robustness analyses that focused on predictors of overall distress response and remission. Distress remission was defined as post-intervention scores within the normal range on all three DASS-21 subscales, and distress response was defined as a ≥ 50% reduction in DASS-21 total score from pre- to post- intervention. These analyses assessed whether the pattern of prognostic and prescriptive predictors differed when modelling overall psychological distress severity as the outcome rather than depressive symptom severity specifically. Consistent with the primary analyses, the overall pattern of predictors was largely similar (see Tables S15–S18), suggesting that several predictors were shared across the two outcome domains. However, several minor differences emerged. For example, more mental health-related GP visits (range = 0–17; OR = 0.80, 95% CI [0.67, 0.96], *p* = 0.019) were significantly associated with lower odds of distress remission, but this association was marginal for depression remission. Baseline depression severity (range = 0–42; OR = 0.98, 95% CI [0.96, 1.01], *p* = 0.159) and pre-treatment credibility (range = 0–17; OR = 1.11, 95% CI [0.99, 1.23], *p* = 0.063) were not significant predictors of distress response, whereas they were associated with depression response. In contrast, higher pre-treatment expectancy (range = 0–17; OR = 1.01, 95% CI [1.00, 1.02], *p* = 0.030) was significantly associated with the odds of distress response, but not depression response. A significant interaction was observed between baseline anxiety severity and the sleep hygiene intervention for distress response (OR = 0.91, 95% CI [0.83, 0.99], *p* = 0.038), indicating lower odds of distress response among participants with higher baseline anxiety. This interaction was not observed for distress remission but was observed for depression outcomes.

## Discussion

This study aimed to identify key prognostic and prescriptive predictors of depression remission and response following brief digital interventions among university students experiencing elevated levels of psychological distress. Using data from a large, AI-adaptive trial, we evaluated a broad range of demographic, clinical, psychosocial, treatment-related, and identity-related predictors. Overall, the findings showed that several baseline characteristics were associated with treatment outcomes.

In terms of prognostic predictors, demographically, neither sex nor SES significantly predicted remission or response, contrary to our hypothesis that higher SES would be associated with better outcomes. Clinically, lower baseline depression severity was consistently linked to a higher likelihood of remission, aligning with expectations^29^. Surprisingly, fewer mental health-related GP visits were associated with higher likelihood of response. Within the psychosocial domain, better mental health-related quality of life predicted higher likelihood of remission, consistent with our hypothesis^46^, while lower perceived social support was a significant predictor of remission only among participants with severe baseline distress. Contrary to our hypothesis, treatment expectancy was not related to outcomes. However, higher perceived treatment credibility was significantly associated with increased response. In the identity domain, no significant effects were observed for both LGBTQA+ status and CALD background. These findings provide partial support for our hypotheses, with baseline depression severity emerging as the most consistent prognostic predictor across outcomes. However, a large proportion of variance in treatment response remained unexplained.

Prescriptive effects were less pronounced. Among all interactions tested, only one prescriptive marker emerged: baseline anxiety severity moderated the effectiveness of the sleep hygiene intervention, aligning with our broader hypothesis that baseline anxiety may function as a prescriptive predictor. Specifically, pairwise analyses indicated that the likelihood of remission and response following sleep hygiene was lower for participants with higher baseline anxiety, relative to those in the physical activity and mindfulness groups. This pattern may indicate relatively more favourable outcomes for physical activity and mindfulness compared to sleep hygiene among individuals with elevated anxiety. By contrast, baseline depression severity and other variables did not significantly moderate treatment outcomes, suggesting that most baseline characteristics did not differentiate treatment effects across the four digital interventions.

In contrast to previous studies that reported inconsistent associations between baseline depression severity and treatment outcomes^32,72^, our findings revealed a clear and consistent pattern: higher baseline severity was associated with lower odds of both remission and response. Although the same set of predictors was examined, model performance differed markedly. Remission was predicted with higher accuracy (Nagelkerke *R*² = 0.34) than response (*R*² = 0.07).

This discrepancy may reflect several possibilities. First, brief interventions may be insufficient for participants with higher baseline severity, whereas longer and more structured approaches, such as internet-delivered CBT, could be more effective for this group. Second, response, defined as a proportional reduction in symptoms, may be more variable and harder to predict, particularly when baseline symptom severity is low. In contrast, remission, defined by reaching a low absolute symptom threshold, may represent a more clinically meaningful and stable endpoint^73,74^. It is less sensitive to fluctuations in baseline severity and may better capture individuals who have achieved a sustained and noticeable recovery^75^. Third, response may be influenced by additional, unmeasured factors not accounted for in current models^76^. For example, defence mechanisms (i.e., coping styles) have been shown to predict response, but not remission^77^. These findings suggest that remission and response may require distinct predictive models^78^. They also point to defining response as a continuous ratio, rather than a binary outcome, to improve its predictive utility^79^.

Higher frequency of mental health-related GP visits was associated with lower odds of response among the full sample. This association—while initially counterintuitive—may reflect underlying illness complexity, with more frequent primary care contact serving as a proxy for more severe, chronic, or comorbid presentations, including comorbid physical illness^80,81^. It also aligns with the idea of a “high utiliser” subgroup, individuals with persistent distress and prior unmet needs who may not respond well to low-intensity interventions^82^.

In the subgroup analyses, baseline depression severity was the only consistent predictor of remission across all distress severity levels, and of response in the moderate and severe groups, with a marginal association in the mild group. More mental health-related GP visits were linked to worse remission and response in the moderate group. In the severe group, higher levels of perceived social support were associated with increased odds of remission.

These findings suggest that baseline depression severity is a key predictor of treatment outcomes, regardless of the initial severity of overall psychological distress. Individuals with moderate distress often exhibit greater variability in symptom profiles and treatment outcomes^53,83,84^. Among individuals with more severe symptoms, perceived social support may play a critical role in facilitating recovery. For example, perceived family support has been found to be negatively associated with suicidal ideation in young people with major depressive disorder^84^. Therefore, when designing digital mental health interventions, it is important to tailor approaches according to different levels of distress. For individuals with severe distress, interventions could be designed to integrate peer-based guidance. The findings also highlight the importance of developing severity-specific predictive models, as treatment outcomes may differ based on individuals’ baseline distress level^76,85^.

Our moderation analyses showed that higher baseline anxiety was associated with lower odds of response and remission in the sleep hygiene condition, relative to the physical activity and mindfulness conditions. Given that anxiety was significantly associated with poorer subjective sleep quality in our sample (Spearman’s *ρ* = −0.16, *p* < 0.001), it is possible that those with more severe anxiety experienced more substantial sleep disturbances that were not adequately addressed by a self-guided sleep hygiene intervention. Prior research supports this association; for example, a study found that anxiety predicted poorer sleep quality, mediated by lower adherence to sleep hygiene behaviours, with *R*² values of 0.27 and 0.22 across two university student samples^86^. Individuals with high baseline anxiety may therefore respond more favourably to sleep interventions that more comprehensively target maladaptive sleep-related cognitions and behaviours, such as CBT for Insomnia (CBT-I)^87,88^. However, these findings should be considered preliminary and require replication in confirmatory trials before informing clinical treatment selection.

This study utilised data from a large-scale, four-arm randomised trial, characterised by clearly defined intervention contrasts and a large sample size. These features provided a strong empirical foundation for examining both prognostic and prescriptive effects. Outcomes from the main trial indicated that the digital interventions produced effects comparable to those observed in studies of face-to-face lifestyle or psychological interventions for university students^89,90^, underscoring their potential applicability within this populations^54^.

However, several limitations should be noted. First, as a secondary analysis, some prognostic variables suggested by previous research, such as symptom duration^29,91^ and the presence of personality disorders^27^ were unavailable. Second, the interventions were originally designed to target general psychological distress rather than depression specifically. However, depression is a core component of distress, and the interventions used are evidence-based treatments for depression^92,93^. Supplementary robustness analyses also demonstrated comparable findings when modelling overall distress outcomes and depression-specific outcomes. Third, although we adjusted for mini-trial number to account for temporal changes in allocation, the response-adaptive design may have reduced observable treatment heterogeneity, potentially attenuating prescriptive effects and, to a lesser extent, influencing estimated treatment and prognostic associations. Fourth, despite the large overall sample, unequal allocation across intervention arms, e.g., the smaller EMA control arm relative to the active treatment arms, may have reduced statistical power for treatment comparisons. Subgroup analyses by baseline severity may have been further underpowered. Fifth, although our definitions align with prior DASS-based trials^94,95^, binary outcomes necessarily involve information loss and may reduce sensitivity to symptom change. The short post-intervention assessment window also limits conclusions regarding the durability of the effects. Finally, in line with previous research, much of the outcome variance remained unexplained^72^, and the predictive utility of models for response was relatively low.

Future research should aim to clarify and standardise the definitions of response and remission to enable comparability of results across studies and enhance the utility of related predictive models. The identification of prognostic and prescriptive factors also requires further replication. Complementary data-driven modelling approaches may help to strengthen and validate emerging findings as larger datasets become available. Emerging research also suggests that trajectory-based approaches, which track early changes over time, may offer a promising avenue for more sensitively identifying prescriptive factors^96^.

Although much of the variance in outcomes remains unexplained, with the best-performing model accounting for 34%, this study highlights the potential role of individual-level factors in shaping depression treatment outcomes to brief digital interventions. Variables such as baseline depression and anxiety severity, as well as mental health-related GP visits and quality of life, may help guide more targeted care. Given that remission and response may reflect distinct clinical processes, separate predictive models may be warranted. The findings also highlight the need for severity-specific approaches and suggest that incorporating trajectory-based measures, which capture early symptom changes over time, may enhance predictive accuracy.

## Supplementary information

Supplementary Tables

CONSORT 2025 checklist_Liu

## Data Availability

The datasets generated and/or analysed during the current study are not publicly available, as the primary trial manuscripts are still under preparation. Data will be available from the corresponding author upon reasonable request and following publication of the primary trial manuscripts.
