# Supplementary material for: Predictors of depression outcomes among university students following brief smartphone-based interventions
Source: Npj Ment Health Res. 2026 Apr 17;5:25. doi: 10.1038/s44184-026-00208-3 (PMC13090371; doi:10.1038/s44184-026-00208-3)
Supplement: Supplementary file 1 — Supplementary Tables [file 44184_2026_208_MOESM1_ESM.pdf]

Predictors of depression outcomes among university students following brief smartphone-based interventions

Xuanchen Liu, M Couns, WuYi Zheng, PhD, Leonard Hoon, PhD, Sunil Gupta, PhD, Svetha Venkatesh, PhD, Helen Christensen, PhD, Jill Newby, PhD, Alexis E. Whitton, PhD on behalf of the *Vibe Up Trial Consortium*.

Supplementary Tables

Table S1: Predictors of Missingness in the Remission Outcome 3

Table S2: Predictors of Missingness in the Response Outcome 4

Table S3: Predictors of Non-Disclosure of LGBTQA+ Status 5

Table S4: Baseline Variables Examined as Candidate Prescriptive Predictors....................... 6

Table S5a: Prognostic Predictors of Remission in the Mild DASS Subgroup 7

Table S5b: Prognostic Predictors of Remission in the Moderate DASS Subgroup 8

Table S5c: Prognostic Predictors of Remission in the Severe DASS Subgroup 9

Table S6a: Prognostic Predictors of Response in the Mild DASS Subgroup 10

Table S6b: Prognostic Predictors of Response in the Moderate DASS Subgroup 11

Table S6c: Prognostic Predictors of Response in the Severe DASS Subgroup 12

Table S7: Full Model Including Treatment × Baseline Anxiety Severity Interaction (Remission Outcome) 13

Table S8: Exploratory Pairwise Treatment Contrasts by Baseline Anxiety Severity (Remission Outcome) 14

Table S9: Full Model Including Treatment × Baseline Anxiety Severity Interaction (Response Outcome) 15

Table S10: Exploratory Pairwise Treatment Contrasts by Baseline Anxiety Severity (Response Outcome) 16

Table S11: Exploratory Interaction Analyses Testing Candidate Prescriptive Predictors (Remission Outcome) 17

Table S12: Exploratory Interaction Analyses Testing Candidate Prescriptive Predictors (Response Outcome) 19

Table S13: Prognostic Predictors of Remission Among Individuals with Elevated Depression 21

Table S14: Full Model Including Treatment × Baseline Anxiety Severity Interaction Among Individuals with Elevated Depression (Remission Outcome) 22

Table S15: Prognostic Predictors of Distress Remission 23

Table S16: Prognostic Predictors of Distress Response 24

Table S17: Full Model Including Treatment × Baseline Anxiety Severity Interaction (Distress Remission Outcome) 25

Table S18: Full Model Including Treatment × Baseline Anxiety Severity Interaction (Distress Response Outcome) 26

Table S1

Predictors of Missingness in the Remission Outcome

Predictor	B (SE)	z value	p-value
Intercept	-1.49 (1.16)	-1.281	.200
Age	-0.05 (0.02)	-1.991	.046
Sex (ref = female)	0.71 (0.23)	3.061	.002
LGBTQA+ Status (ref = non-LGBTQA+)	0.09 (0.22)	0.406	.684
CALD Background (ref = non-CALD)	-0.60 (0.23)	-2.600	.009
Socioeconomic Status	0.08 (0.06)	1.300	.194
Baseline Depression Severity	0.02 (0.01)	1.220	.223
Perceived Social Support	0.12 (0.11)	1.033	.301
Recovering Quality of Life	-0.01 (0.03)	-0.471	.637
GP Visit Frequency (Past 12 Weeks)	0.01 (0.09)	0.155	.877
Treatment Credibility	-0.08 (0.07)	-1.122	.262
Treatment Expectancy	-0.01 (0.01)	-1.072	.284

Note. Logistic regression model predicting whether remission outcome data were missing (1 = missing, 0 = observed). Significant associations with observed variables (e.g., age, sex, CALD background) support the assumption that data were MAR. B = unstandardised logistic regression coefficient (log odds); SE = standard error.

Table S2
Predictors of Missingness in the Response Outcome

Predictor	B (SE)	z value	p-value
Intercept	-1.72 (1.15)	-1.500	.134
Age	-0.04 (0.02)	-1.746	.081
Sex (ref = female)	0.69 (0.23)	2.990	.003
LGBTQA+ Status (ref = non-LGBTQA+)	0.15 (0.22)	0.681	.496
CALD Background (ref = non-CALD)	-0.61 (0.23)	-2.690	.007
Socioeconomic Status	0.08 (0.06)	1.292	.196
Baseline Depression Severity	0.02 (0.01)	1.034	.301
Perceived Social Support	0.11 (0.11)	0.953	.341
Recovering Quality of Life	-0.01 (0.02)	-0.225	.822
GP Visit Frequency (Past 12 Weeks)	0.04 (0.09)	0.418	.676
Treatment Credibility	-0.07 (0.07)	-0.971	.332
Treatment Expectancy	-0.01 (0.01)	-1.284	.199

Note. Logistic regression model predicting whether response outcome data were missing (1 = missing, 0 = observed). Significant associations with observed variables (e.g., sex, CALD background) support the assumption that data were MAR. B = unstandardised logistic regression coefficient (log odds); SE = standard error.

Table S3

Predictors of Non-Disclosure of LGBTQA+ Status

Predictor	B (SE)	z value	p-value
Intercept	-1.38 (1.28)	-1.075	.282
Age	-0.03 (0.03)	-1.189	.234
Sex (ref = female)	-0.53 (0.37)	-1.428	.153
Baseline Depression Severity	-0.00 (0.02)	-0.261	.794
Treatment Credibility	0.06 (0.08)	0.731	.465
Recovering Quality of Life	-0.04 (0.03)	-1.281	.200

Note. Logistic regression model predicting whether participants did not disclose their LGBTQA+ status (1 = 'prefer not to say' or 'don't know'; 0 = disclosed). No predictors were significantly associated with non-disclosure at the $p < .05$ level, suggesting no substantial bias in the missingness pattern. B = unstandardised logistic regression coefficient (log odds); SE = standard error.

Table S4

Baseline Variables Examined as Candidate Prescriptive Predictors

Predictor	Measurement	Type
Age	Self-reported age in years	Demographic
Socioeconomic Status	Self-rated socioeconomic status on a 10-point scale	Demographic
Employment Status	Self-reported paid employment in the past 12 weeks (Yes/No)	Demographic
Baseline Distress Severity	Total score on the Depression Anxiety Stress Scales-21 (DASS-21)	Clinical
Baseline Anxiety Severity	Anxiety subscale score of the DASS-21	Clinical
Baseline Depression Severity	Depression subscale score of the DASS-21	Clinical
Mental Health Service Use	Frequency of general practitioner (GP) visits for mental health concerns in the past 12 weeks	Clinical
Multiple Diagnosis	Self-reported diagnosis of more than one mental health condition (Yes/No)	Clinical
Readiness to Change	Total score on the abridged Revised University of Rhode Island Change Assessment Scale	Psychosocial
Helplessness	Agreement with the statement “I thought my life was not worth living” rated on a 5-point scale	Psychosocial
LGBTQA+ Status	Sexual minority status (Yes if participants selected “Gay or lesbian”, “Bisexual”, or “I use a different term”; No otherwise)	Identity-related
CALD Background	Culturally and linguistically diverse background (Yes if participants reported speaking a language other than English at home or an ancestry other than Australian, British, or Irish; No otherwise)	Identity-related

Note. Prescriptive predictors were examined by including interaction terms between each baseline variable and treatment condition in hierarchical logistic regression models.

Table S5a

Prognostic Predictors of Remission in the Mild DASS Subgroup

Predictor	B (SE)	OR [95% CI]	p-value
Sex (ref = female)	-0.20 (0.26)	0.82 [0.49, 1.37]	.453
Socioeconomic Status	0.05 (0.07)	1.05 [0.92, 1.19]	.465
Baseline Depression Severity	-0.12 (0.02)	0.88 [0.84, 0.92]	<.001
Perceived Social Support	0.09 (0.12)	1.09 [0.87, 1.38]	.448
Recovering Quality of Life	0.06 (0.03)	1.06 [1.00, 1.12]	.055
GP Visit Frequency	-0.02 (0.14)	0.98 [0.74, 1.29]	.880
Treatment Credibility	0.04 (0.08)	1.04 [0.90, 1.21]	.566
Treatment Expectancy	0.00 (0.01)	1.00 [0.99, 1.02]	.529
LGBTQA+ Status (ref = non-LGBTQA+)	-0.40 (0.24)	0.67 [0.42, 1.07]	.096
CALD Background (ref = non-CALD)	0.15 (0.22)	1.16 [0.76, 1.79]	.492

Note. B = unstandardised logistic regression coefficient (log odds); SE = standard error; OR = odds ratio; CI = confidence interval.

Table S5b

Prognostic Predictors of Remission in the Moderate DASS Subgroup

Predictor	B (SE)	OR [95% CI]	p-value
Sex (ref = female)	-0.38 (0.35)	0.68 [0.34, 1.36]	.277
Socioeconomic Status	-0.03 (0.07)	0.97 [0.84, 1.12]	.681
Baseline Depression Severity	-0.13 (0.03)	0.88 [0.83, 0.92]	<.001
Perceived Social Support	0.02 (0.13)	1.02 [0.80, 1.31]	.862
Recovering Quality of Life	0.05 (0.03)	1.06 [0.99, 1.12]	.080
GP Visit Frequency (Past 12 Weeks)	-0.29 (0.13)	0.75 [0.59, 0.96]	.024
Treatment Credibility	0.01 (0.09)	1.01 [0.86, 1.20]	.870
Treatment Expectancy	0.01 (0.01)	1.01 [0.99, 1.02]	.442
LGBTQA+ Status (ref = non-LGBTQA+)	-0.26 (0.26)	0.77 [0.47, 1.29]	.327
CALD Background (ref = non-CALD)	0.33 (0.25)	1.39 [0.85, 2.30]	.194

Note. B = unstandardised logistic regression coefficient (log odds); SE = standard error; OR = odds ratio; CI = confidence interval.

Table S5c

Prognostic Predictors of Remission in the Severe DASS Subgroup

Predictor	B (SE)	OR [95% CI]	p-value
Sex (ref = female)	0.11 (0.47)	1.11 [0.45, 2.77]	.820
Socioeconomic Status	-0.04 (0.09)	0.96 [0.80, 1.16]	.681
Baseline Depression Severity	-0.12 (0.03)	0.89 [0.84, 0.95]	<.001
Perceived Social Support	0.35 (0.17)	1.42 [1.02, 1.97]	.039
Recovering Quality of Life	0.04 (0.04)	1.04 [0.97, 1.12]	.269
GP Visit Frequency (Past 12 Weeks)	-0.08 (0.10)	0.93 [0.76, 1.13]	.448
Treatment Credibility	0.06 (0.12)	1.06 [0.84, 1.33]	.621
Treatment Expectancy	0.01 (0.01)	1.01 [0.99, 1.03]	.181
LGBTQA+ Status (ref = non-LGBTQA+)	-0.21 (0.35)	0.81 [0.41, 1.62]	.554
CALD Background (ref = non-CALD)	-0.05 (0.37)	0.95 [0.46, 1.95]	.890

Note. B = unstandardised logistic regression coefficient (log odds); SE = standard error; OR = odds ratio; CI = confidence interval.

Table S6a

Prognostic Predictors of Response in the Mild DASS Subgroup

Predictor	B (SE)	OR [95% CI]	p-value
Sex (ref = female)	0.25 (0.24)	1.29 [0.80, 2.06]	.297
Socioeconomic Status	0.02 (0.06)	1.02 [0.90, 1.15]	.787
Baseline Depression Severity	-0.05 (0.02)	0.95 [0.91, 0.99]	.022
Perceived Social Support	-0.01 (0.11)	0.99 [0.80, 1.23]	.937
Recovering Quality of Life	-0.01 (0.03)	0.99 [0.94, 1.05]	.796
GP Visit Frequency (Past 12 Weeks)	-0.07 (0.14)	0.93 [0.71, 1.21]	.590
Treatment Credibility	0.07 (0.07)	1.07 [0.93, 1.23]	.339
Treatment Expectancy	-0.00 (0.01)	1.00 [0.99, 1.01]	.731
LGBTQA+ Status (ref = non-LGBTQA+)	0.09 (0.22)	1.09 [0.71, 1.68]	.696
CALD Background (ref = non-CALD)	-0.15 (0.21)	0.86 [0.57, 1.30]	.479

Note. B = unstandardised logistic regression coefficient (log odds); SE = standard error; OR = odds ratio; CI = confidence interval.

Table S6b

Prognostic Predictors of Response in the Moderate DASS Subgroup

Predictor	B (SE)	OR [95% CI]	p-value
Sex (ref = female)	-0.21 (0.35)	0.81 [0.41, 1.61]	.555
Socioeconomic Status	0.01 (0.07)	1.01 [0.87, 1.16]	.941
Baseline Depression Severity	-0.06 (0.02)	0.94 [0.90, 0.99]	.015
Perceived Social Support	0.13 (0.13)	1.14 [0.88, 1.48]	.319
Recovering Quality of Life	-0.02 (0.03)	0.98 [0.92, 1.04]	.419
GP Visit Frequency (Past 12 Weeks)	-0.33 (0.14)	0.72 [0.55, 0.94]	.016
Treatment Credibility	0.10 (0.09)	1.11 [0.93, 1.32]	.254
Treatment Expectancy	-0.00 (0.01)	1.00 [0.99, 1.01]	.946
LGBTQA+ Status (ref = non-LGBTQA+)	-0.27 (0.28)	0.76 [0.44, 1.31]	.327
CALD Background (ref = non-CALD)	0.12 (0.25)	1.13 [0.69, 1.86]	.631

Note. B = unstandardised logistic regression coefficient (log odds); SE = standard error; OR = odds ratio; CI = confidence interval.

Table S6c

Prognostic Predictors of Response in the Severe DASS Subgroup

Predictor	B (SE)	OR [95% CI]	p-value
Sex (ref = female)	0.12 (0.39)	1.13 [0.53, 2.41]	.749
Socioeconomic Status	-0.03 (0.09)	0.97 [0.81, 1.17]	.765
Baseline Depression Severity	-0.06 (0.02)	0.95 [0.91, 0.99]	.016
Perceived Social Support	0.14 (0.13)	1.16 [0.88, 1.52]	.283
Recovering Quality of Life	0.01 (0.03)	1.01 [0.94, 1.07]	.824
GP Visit Frequency (Past 12 Weeks)	-0.06 (0.09)	0.95 [0.79, 1.15]	.533
Treatment Credibility	0.16 (0.10)	1.17 [0.96, 1.42]	.128
Treatment Expectancy	0.01 (0.01)	1.01 [0.99, 1.03]	.365
LGBTQA+ Status (ref = non-LGBTQA+)	0.15 (0.29)	1.16 [0.66, 2.04]	.604
CALD Background (ref = non-CALD)	0.15 (0.28)	1.16 [0.67, 2.01]	.593

Note. B = unstandardised logistic regression coefficient (log odds); SE = standard error; OR = odds ratio; CI = confidence interval.

Table S7

Full Model Including Treatment × Baseline Anxiety Severity Interaction (Remission Outcome)

Predictor	B (SE)	OR [95% CI]	p-value
Treatment: Sleep Hygiene	1.11 (0.55)	3.02 [1.04, 8.83]	.043
Treatment: Mindfulness	0.33 (0.57)	1.38 [0.45, 4.25]	.570
Treatment: Physical Activity	0.34 (0.54)	1.40 [0.48, 4.05]	.534
Baseline Anxiety Severity	0.04 (0.03)	1.04 [0.97, 1.11]	.248
Mini-trial Number	0.03 (0.02)	1.04 [0.99, 1.08]	.113
Sex (ref = female)	-0.19 (0.19)	0.83 [0.57, 1.20]	.327
Socioeconomic Status	-0.00 (0.04)	1.00 [0.92, 1.09]	.962
Baseline Depression Severity	-0.12 (0.01)	0.88 [0.86, 0.91]	<.001
Perceived Social Support	0.12 (0.07)	1.13 [0.98, 1.30]	.102
Recovering Quality of Life	0.05 (0.02)	1.05 [1.02, 1.09]	.006
GP Visit Frequency (Past 12 Weeks)	-0.14 (0.07)	0.87 [0.76, 1.00]	.056
Treatment Credibility	0.04 (0.05)	1.04 [0.94, 1.15]	.420
Treatment Expectancy	0.01 (0.00)	1.01 [1.00, 1.01]	.148
LGBTQA+ Status (ref = non-LGBTQA+)	-0.29 (0.16)	0.75 [0.55, 1.02]	.068
CALD Background (ref = non-CALD)	0.17 (0.15)	1.18 [0.88, 1.59]	.261
Sleep Hygiene × Baseline Anxiety	-0.11 (0.04)	0.90 [0.83, 0.97]	.009
Mindfulness × Baseline Anxiety	-0.03 (0.04)	0.97 [0.90, 1.05]	.483
Physical Activity × Baseline Anxiety	-0.01 (0.04)	0.99 [0.92, 1.07]	.796

Note. B = unstandardised logistic regression coefficient (log odds); SE = standard error; OR = odds ratio; CI = confidence interval.

Table S8

Exploratory Pairwise Treatment Contrasts by Baseline Anxiety Severity (Remission Outcome)

Baseline Anxiety	Contrast	B (SE)	OR [95% CI]	p-value
Low (5.09)	EMA vs Sleep Hygiene	-0.57 (0.40)	0.57 [0.26, 1.23]	.149
	EMA vs Mindfulness	-0.19 (0.42)	0.83 [0.36, 1.90]	.655
	EMA vs Physical Activity	-0.29 (0.40)	0.75 [0.34, 1.65]	.474
	Sleep Hygiene vs Mindfulness	0.38 (0.27)	1.46 [0.86, 2.48]	.155
	Sleep Hygiene vs Physical Activity	0.28 (0.24)	1.33 [0.83, 2.12]	.238
	Mindfulness vs Physical Activity	-0.10 (0.28)	0.91 [0.52, 1.58]	.727
Moderate (12.67)	EMA vs Sleep Hygiene	-0.57 (0.40)	0.57 [0.26, 1.23]	.149
	EMA vs Mindfulness	-0.19 (0.42)	0.83 [0.36, 1.90]	.655
	EMA vs Physical Activity	-0.29 (0.40)	0.75 [0.34, 1.65]	.474
	Sleep Hygiene vs Mindfulness	0.38 (0.27)	1.46 [0.86, 2.48]	.155
	Sleep Hygiene vs Physical Activity	0.28 (0.24)	1.33 [0.83, 2.12]	.238
	Mindfulness vs Physical Activity	-0.10 (0.28)	0.91 [0.52, 1.58]	.727
High (20.24)	EMA vs Sleep Hygiene	1.03 (0.48)	2.79 [1.10, 7.10]	.031
	EMA vs Mindfulness	0.22 (0.43)	1.24 [0.54, 2.88]	.614
	EMA vs Physical Activity	-0.14 (0.45)	0.87 [0.36, 2.09]	.755
	Sleep Hygiene vs Mindfulness	-0.81 (0.32)	0.44 [0.24, 0.83]	.011
	Sleep Hygiene vs Physical Activity	-1.17 (0.35)	0.31 [0.16, 0.62]	< .001
	Mindfulness vs Physical Activity	-0.36 (0.27)	0.70 [0.41, 1.20]	.194

Note. Estimates are derived from the prescriptive interaction model including treatment condition, baseline anxiety severity, and their interaction. Baseline anxiety values represent the mean and ±1 SD.

Table S9

Full Model Including Treatment × Baseline Anxiety Severity Interaction (Response Outcome)

Predictor	B (SE)	OR [95% CI]	p-value
Treatment: Sleep Hygiene	1.18 (0.58)	3.27 [1.05, 10.21]	.042
Treatment: Mindfulness	0.58 (0.59)	1.78 [0.56, 5.66]	.328
Treatment: Physical Activity	0.46 (0.58)	1.58 [0.50, 4.96]	.433
Baseline Anxiety Severity	0.03 (0.03)	1.03 [0.96, 1.10]	.392
Mini-trial Number	0.03 (0.02)	1.03 [0.99, 1.07]	.172
Sex (ref = female)	0.09 (0.18)	1.09 [0.77, 1.55]	.614
Socioeconomic Status	-0.00 (0.04)	1.00 [0.92, 1.08]	.992
Baseline Depression Severity	-0.05 (0.01)	0.95 [0.93, 0.97]	<.001
Perceived Social Support	0.09 (0.07)	1.09 [0.95, 1.25]	.209
Recovering Quality of Life	-0.01 (0.02)	0.99 [0.96, 1.02]	.448
GP Visit Frequency (Past 12 Weeks)	-0.15 (0.07)	0.86 [0.76, 0.99]	.032
Treatment Credibility	0.10 (0.05)	1.11 [1.01, 1.22]	.029
Treatment Expectancy	0.00 (0.00)	1.00 [0.99, 1.01]	.812
LGBTQA+ Status (ref = non-LGBTQA+)	-0.02 (0.15)	0.98 [0.74, 1.31]	.907
CALD Background (ref = non-CALD)	0.00 (0.14)	1.00 [0.76, 1.32]	.990
Sleep Hygiene × Baseline Anxiety	-0.07 (0.04)	0.93 [0.86, 1.00]	.061
Mindfulness × Baseline Anxiety	-0.00 (0.04)	1.00 [0.93, 1.07]	.975
Physical Activity × Baseline Anxiety	0.02 (0.04)	1.02 [0.95, 1.10]	.551

Note. B = unstandardised logistic regression coefficient (log odds); SE = standard error; OR = odds ratio; CI = confidence interval.

Table S10

Exploratory Pairwise Treatment Contrasts by Baseline Anxiety Severity (Response Outcome)

Baseline Anxiety	Contrast	B (SE)	OR [95% CI]	p-value
Low (5.09)	EMA vs Sleep Hygiene	-0.81 (0.43)	0.44 [0.19, 1.04]	.062
	EMA vs Mindfulness	-0.57 (0.45)	0.56 [0.23, 1.36]	.202
	EMA vs Physical Activity	-0.57 (0.44)	0.57 [0.24, 1.34]	.197
	Sleep Hygiene vs Mindfulness	0.24 (0.24)	1.27 [0.79, 2.05]	.327
	Sleep Hygiene vs Physical Activity	0.24 (0.23)	1.27 [0.81, 2.00]	.295
	Mindfulness vs Physical Activity	0.00 (0.27)	1.00 [0.59, 1.70]	.994
Moderate (12.67)	EMA vs Sleep Hygiene	-0.26 (0.33)	0.77 [0.40, 1.49]	.443
	EMA vs Mindfulness	-0.56 (0.33)	0.57 [0.30, 1.09]	.088
	EMA vs Physical Activity	-0.74 (0.33)	0.48 [0.25, 0.91]	.025
	Sleep Hygiene vs Mindfulness	-0.31 (0.19)	0.74 [0.51, 1.06]	.100
	Sleep Hygiene vs Physical Activity	-0.48 (0.19)	0.62 [0.43, 0.89]	.010
	Mindfulness vs Physical Activity	-0.17 (0.18)	0.84 [0.59, 1.20]	.340
High (20.24)	EMA vs Sleep Hygiene	0.30 (0.46)	1.35 [0.55, 3.31]	.514
	EMA vs Mindfulness	-0.55 (0.41)	0.57 [0.26, 1.28]	.177
	EMA vs Physical Activity	-0.90 (0.42)	0.41 [0.18, 0.93]	.032
	Sleep Hygiene vs Mindfulness	-0.85 (0.29)	0.43 [0.24, 0.76]	.004
	Sleep Hygiene vs Physical Activity	-1.20 (0.31)	0.30 [0.16, 0.55]	< .001
	Mindfulness vs Physical Activity	-0.35 (0.23)	0.71 [0.45, 1.11]	.132

Note. Estimates are derived from the prescriptive interaction model including treatment condition, baseline anxiety severity, and their interaction. Baseline anxiety values represent the mean and ±1 SD.

Table S11

Exploratory Interaction Analyses Testing Candidate Prescriptive Predictors (Remission Outcome)

Predictor	Interaction Term	B (SE)	OR [95% CI]	p-value
Age	Sleep Hygiene × Age	-0.09 (0.07)	0.91 [0.79, 1.05]	.212
	Mindfulness × Age	-0.05 (0.07)	0.95 [0.82, 1.10]	.485
	Physical Activity × Age	-0.08 (0.07)	0.93 [0.80, 1.07]	.296
Socioeconomic Status	Sleep Hygiene × Socioeconomic Status	0.27 (0.16)	1.31 [0.95, 1.79]	.099
	Mindfulness × Socioeconomic Status	0.18 (0.17)	1.20 [0.87, 1.66]	.265
	Physical Activity × Socioeconomic Status	0.15 (0.17)	1.17 [0.84, 1.62]	.358
Employment Status	Sleep Hygiene × Employment	0.66 (0.71)	1.93 [0.48, 7.71]	.354
	Mindfulness × Employment	0.65 (0.72)	1.91 [0.47, 7.84]	.369
	Physical Activity × Employment	0.45 (0.73)	1.56 [0.37, 6.56]	.541
Baseline Distress Severity	Sleep Hygiene × Distress	-0.03 (0.02)	0.97 [0.94, 1.00]	.080
	Mindfulness × Distress	0.00 (0.02)	1.00 [0.97, 1.03]	.788
	Physical Activity × Distress	0.00 (0.01)	1.00 [0.97, 1.03]	.855
Baseline Depression Severity	Sleep Hygiene × Depression	-0.02 (0.04)	0.98 [0.90, 1.07]	.629
	Mindfulness × Depression	0.02 (0.04)	1.02 [0.95, 1.11]	.543
	Physical Activity × Depression	0.00 (0.04)	1.00 [0.92, 1.09]	.977
Mental Health Service Use	Sleep Hygiene × Service Use	0.11 (0.28)	1.12 [0.65, 1.93]	.682
	Mindfulness × Service Use	0.12 (0.27)	1.13 [0.67, 1.90]	.656
	Physical Activity × Service Use	0.20 (0.28)	1.22 [0.71, 2.10]	.475
Multiple Diagnosis	Sleep Hygiene × Multiple Diagnosis	-0.19 (0.65)	0.83 [0.23, 2.96]	.775
	Mindfulness × Multiple Diagnosis	-0.10 (0.65)	0.91 [0.25, 3.25]	.881
	Physical Activity × Multiple Diagnosis	-0.19 (0.67)	0.83 [0.22, 3.06]	.777
Readiness to Change	Sleep Hygiene × Readiness to Change	-0.06 (0.11)	0.95 [0.77, 1.17]	.609
	Mindfulness × Readiness to Change	-0.05 (0.11)	0.95 [0.76, 1.18]	.636
	Physical Activity × Readiness to Change	0.02 (0.12)	1.02 [0.81, 1.28]	.883
Helplessness	Sleep Hygiene × Helplessness	0.45 (0.37)	1.57 [0.76, 3.25]	.224
	Mindfulness × Helplessness	0.28 (0.36)	1.32 [0.65, 2.67]	.446
	Physical Activity × Helplessness	0.39 (0.40)	1.48 [0.68, 3.22]	.318

Predictor	Interaction Term	B (SE)	OR [95% CI]	p-value
LGBTQA+ Status	Sleep Hygiene × LGBTQA+	-0.87 (0.62)	0.42 [0.13, 1.40]	.159
	Mindfulness × LGBTQA+	-0.99 (0.63)	0.37 [0.11, 1.27]	.115
	Physical Activity × LGBTQA+	-0.96 (0.64)	0.38 [0.11, 1.34]	.133
CALD Background	Sleep Hygiene × CALD	-0.28 (0.60)	0.76 [0.23, 2.46]	.641
	Mindfulness × CALD	-0.09 (0.61)	0.91 [0.28, 3.02]	.882
	Physical Activity × CALD	-0.17 (0.62)	0.84 [0.25, 2.86]	.786

Note. All models were adjusted for trial, sex, socioeconomic status, baseline depression severity, perceived social support, quality of life, mental health service use, treatment credibility, treatment expectancy, LGBTQA+ status and CALD background. B = unstandardised logistic regression coefficient (log odds); SE = standard error; OR = odds ratio; CI = confidence interval. All treatment effects are reported relative to the EMA condition.

Table S12

Exploratory Interaction Analyses Testing Candidate Prescriptive Predictors (Response Outcome)

Predictor	Interaction Term	B (SE)	OR [95% CI]	p-value
Age	Sleep Hygiene × Age	-0.02 (0.07)	0.98 [0.85, 1.13]	.779
	Mindfulness × Age	-0.04 (0.07)	0.96 [0.83, 1.11]	.555
	Physical Activity × Age	-0.04 (0.07)	0.96 [0.83, 1.11]	.574
Socioeconomic Status	Sleep Hygiene × Socioeconomic Status	-0.05 (0.17)	0.95 [0.67, 1.34]	.773
	Mindfulness × Socioeconomic Status	-0.16 (0.18)	0.85 [0.60, 1.21]	.374
	Physical Activity × Socioeconomic Status	-0.11 (0.18)	0.90 [0.64, 1.28]	.554
Employment Status	Sleep Hygiene × Employment	0.79 (0.74)	2.20 [0.52, 9.36]	.288
	Mindfulness × Employment	0.28 (0.74)	1.32 [0.31, 5.60]	.702
	Physical Activity × Employment	0.17 (0.74)	1.18 [0.28, 5.02]	.822
Baseline Distress Severity	Sleep Hygiene × Distress	-0.01 (0.01)	0.99 [0.96, 1.02]	.431
	Mindfulness × Distress	0.01 (0.01)	1.01 [0.98, 1.04]	.533
	Physical Activity × Distress	0.02 (0.01)	1.02 [0.99, 1.05]	.203
Baseline Depression Severity	Sleep Hygiene × Depression	-0.00 (0.04)	1.00 [0.93, 1.07]	.922
	Mindfulness × Depression	0.02 (0.04)	1.02 [0.95, 1.09]	.585
	Physical Activity × Depression	0.04 (0.04)	1.04 [0.97, 1.12]	.294
Mental Health Service Use	Sleep Hygiene × Service Use	-0.05 (0.30)	0.95 [0.53, 1.70]	.869
	Mindfulness × Service Use	0.10 (0.28)	1.10 [0.64, 1.91]	.730
	Physical Activity × Service Use	0.22 (0.29)	1.25 [0.71, 2.20]	.434
Multiple Diagnosis	Sleep Hygiene × Multiple Diagnosis	-0.67 (0.68)	0.51 [0.14, 1.93]	.324
	Mindfulness × Multiple Diagnosis	-0.39 (0.67)	0.68 [0.18, 2.53]	.561
	Physical Activity × Multiple Diagnosis	-0.44 (0.68)	0.64 [0.17, 2.43]	.515
Readiness to Change	Sleep Hygiene × Readiness to Change	-0.02 (0.12)	0.98 [0.77, 1.24]	.848
	Mindfulness × Readiness to Change	-0.03 (0.12)	0.97 [0.77, 1.23]	.796
	Physical Activity × Readiness to Change	0.03 (0.13)	1.03 [0.79, 1.34]	.831
Helplessness	Sleep Hygiene × Helplessness	0.12 (0.40)	1.13 [0.52, 2.45]	.755
	Mindfulness × Helplessness	-0.24 (0.38)	0.79 [0.38, 1.66]	.533
	Physical Activity × Helplessness	-0.25 (0.40)	0.78 [0.36, 1.70]	.530

Predictor	Interaction Term	B (SE)	OR [95% CI]	p-value
LGBTQA+ Status	Sleep Hygiene × LGBTQA+	-0.73 (0.67)	0.48 [0.13, 1.78]	.272
	Mindfulness × LGBTQA+	-0.70 (0.67)	0.50 [0.13, 1.86]	.299
	Physical Activity × LGBTQA+	-0.58 (0.67)	0.56 [0.15, 2.09]	.389
CALD Background	Sleep Hygiene × CALD	-0.33 (0.64)	0.72 [0.20, 2.53]	.609
	Mindfulness × CALD	-0.23 (0.64)	0.79 [0.23, 2.79]	.719
	Physical Activity × CALD	0.00 (0.65)	1.00 [0.28, 3.57]	.999

Note. All models were adjusted for trial, sex, socioeconomic status, baseline depression severity, perceived social support, quality of life, mental health service use, treatment credibility, treatment expectancy, LGBTQA+ status and CALD background. B = unstandardised logistic regression coefficient (log odds); SE = standard error; OR = odds ratio; CI = confidence interval. All treatment effects are reported relative to the EMA condition.

Table S13

Prognostic Predictors of Remission Among Individuals with Elevated Depression

	B (SE)	OR [95% CI]	p-value
**Model 1**			
Sex (ref = female)	-0.19 (0.18)	0.82 [0.57, 1.18]	.291
Socioeconomic Status	0.09 (0.04)	1.10 [1.01, 1.19]	.027
**Model 2**			
Sex (ref = female)	-0.09 (0.20)	0.91 [0.61, 1.36]	.658
Socioeconomic Status	-0.02 (0.05)	0.98 [0.90, 1.08]	.744
Baseline Depression Severity	-0.11 (0.02)	0.89 [0.87, 0.92]	< .001
Perceived Social Support	0.09 (0.08)	1.10 [0.93, 1.29]	.258
Recovering Quality of Life	0.06 (0.02)	1.07 [1.02, 1.11]	.002
GP Visit Frequency (Past 12 Weeks)	-0.14 (0.08)	0.87 [0.75, 1.00]	.058
**Model 3**			
Sex (ref = female)	-0.15 (0.21)	0.86 [0.57, 1.30]	.479
Socioeconomic Status	-0.02 (0.05)	0.98 [0.89, 1.07]	.632
Baseline Depression Severity	-0.11 (0.02)	0.89 [0.86, 0.92]	< .001
Perceived Social Support	0.11 (0.08)	1.11 [0.95, 1.31]	.194
Recovering Quality of Life	0.06 (0.02)	1.06 [1.02, 1.10]	.004
GP Visit Frequency (Past 12 Weeks)	-0.13 (0.08)	0.88 [0.75, 1.02]	.081
Treatment Credibility	0.04 (0.06)	1.04 [0.93, 1.17]	.467
Treatment Expectancy	0.01 (0.00)	1.01 [1.00, 1.02]	.168
LGBTQA+ Status (ref = non-LGBTQA+)	-0.29 (0.18)	0.75 [0.53, 1.06]	.106
CALD Background (ref = non-CALD)	0.12 (0.17)	1.13 [0.81, 1.57]	.466

Note. All models were estimated using hierarchical logistic regression with multiple imputation. Treatment condition and trial number were included as control variables in all models but are not shown in the table. The analytic sample was restricted to participants with at least mild baseline depressive symptoms (DASS-21 depression > 9). B = log-odds coefficient; SE = standard error; OR = odds ratio; CI = confidence interval.

Table S14

Full Model Including Treatment × Baseline Anxiety Severity Interaction Among Individuals with Elevated Depression (Remission Outcome)

Predictor	B (SE)	OR [95% CI]	p-value
Treatment: Sleep Hygiene	2.47 (0.85)	11.83 [2.11, 66.38]	.004
Treatment: Mindfulness	1.30 (0.88)	3.67 [0.68, 19.91]	.143
Treatment: Physical Activity	1.51 (0.82)	4.54 [0.91, 22.59]	.066
Baseline Anxiety Severity	0.09 (0.04)	1.09 [1.01, 1.18]	.039
Mini-trial Number	0.00 (0.03)	1.00 [0.95, 1.05]	.957
Sex (ref = female)	-0.19 (0.21)	0.83 [0.55, 1.26]	.363
Socioeconomic Status	-0.03 (0.05)	0.97 [0.88, 1.06]	.475
Baseline Depression Severity	-0.12 (0.02)	0.89 [0.86, 0.93]	<.001
Perceived Social Support	0.09 (0.09)	1.10 [0.91, 1.32]	.322
Recovering Quality of Life	0.07 (0.03)	1.07 [1.01, 1.15]	.027
GP Visit Frequency (Past 12 Weeks)	-0.13 (0.08)	0.88 [0.75, 1.03]	.099
Treatment Credibility	0.06 (0.07)	1.06 [0.91, 1.24]	.399
Treatment Expectancy	0.01 (0.01)	1.01 [0.99, 1.02]	.147
LGBTQA+ Status (ref = non-LGBTQA+)	-0.29 (0.17)	0.75 [0.54, 1.05]	.078
CALD Background (ref = non-CALD)	0.15 (0.17)	1.16 [0.84, 1.61]	.378
Sleep Hygiene × Baseline Anxiety	-0.17 (0.05)	0.84 [0.76, 0.93]	.001
Mindfulness × Baseline Anxiety	-0.07 (0.05)	0.93 [0.84, 1.04]	.172
Physical Activity × Baseline Anxiety	-0.06 (0.05)	0.94 [0.85, 1.05]	.186

Note. The analytic sample was restricted to participants with at least mild baseline depressive symptoms (DASS-21 depression > 9). B = unstandardised logistic regression coefficient (log odds); SE = standard error; OR = odds ratio; CI = confidence interval.

Table S15
Prognostic Predictors of Distress Remission

	B (SE)	OR [95% CI]	p-value
**Model 1**			
Sex (ref = female)	0.05 (0.18)	1.05 [0.74, 1.49]	.774
Socioeconomic Status	0.10 (0.04)	1.11 [1.02, 1.21]	.013
**Model 2**			
Sex (ref = female)	0.16 (0.19)	1.17 [0.80, 1.71]	.420
Socioeconomic Status	0.00 (0.05)	1.00 [0.91, 1.10]	.946
Baseline Depression Severity	-0.10 (0.01)	0.91 [0.88, 0.93]	< .001
Perceived Social Support	-0.11 (0.08)	0.90 [0.77, 1.06]	.196
Recovering Quality of Life	0.05 (0.02)	1.05 [1.01, 1.09]	.017
GP Visit Frequency (Past 12 Weeks)	-0.22 (0.09)	0.80 [0.67, 0.96]	.017
**Model 3**			
Sex (ref = female)	0.12 (0.20)	1.13 [0.77, 1.66]	.537
Socioeconomic Status	-0.01 (0.05)	0.99 [0.90, 1.09]	.863
Baseline Depression Severity	-0.10 (0.01)	0.91 [0.88, 0.93]	< .001
Perceived Social Support	-0.10 (0.08)	0.90 [0.77, 1.06]	.217
Recovering Quality of Life	0.04 (0.02)	1.04 [1.00, 1.08]	.033
GP Visit Frequency (Past 12 Weeks)	-0.22 (0.09)	0.80 [0.67, 0.96]	.019
Treatment Credibility	0.08 (0.05)	1.09 [0.98, 1.21]	.128
Treatment Expectancy	0.01 (0.00)	1.01 [1.00, 1.01]	.222
LGBTQA+ Status (ref = non-LGBTQA+)	-0.09 (0.17)	0.91 [0.65, 1.28]	.596
CALD Background (ref = non-CALD)	0.02 (0.16)	1.02 [0.74, 1.40]	.916

Note. Treatment condition and mini-trial number were included in the model as control variables but are not interpreted here. The intercept is omitted. B = unstandardised logistic regression coefficient (log odds); SE = standard error; OR = odds ratio; CI = confidence interval.

Table S16
Prognostic Predictors of Distress Response

	B (SE)	OR [95% CI]	p-value
**Model 1**			
Sex (ref = female)	0.05 (0.18)	1.05 [0.74, 1.50]	.787
Socioeconomic Status	0.01 (0.04)	1.01 [0.93, 1.09]	.853
**Model 2**			
Sex (ref = female)	0.07 (0.19)	1.07 [0.75, 1.54]	.700
Socioeconomic Status	-0.03 (0.04)	0.97 [0.88, 1.05]	.434
Baseline Depression Severity	-0.02 (0.01)	0.98 [0.96, 1.00]	.131
Perceived Social Support	0.11 (0.08)	1.11 [0.96, 1.30]	.165
Recovering Quality of Life	0.00 (0.02)	1.00 [0.97, 1.04]	.938
GP Visit Frequency (Past 12 Weeks)	-0.20 (0.08)	0.82 [0.70, 0.96]	.014
**Model 3**			
Sex (ref = female)	0.03 (0.19)	1.03 [0.71, 1.49]	.879
Socioeconomic Status	-0.04 (0.05)	0.96 [0.87, 1.05]	.330
Baseline Depression Severity	-0.02 (0.01)	0.98 [0.96, 1.01]	.159
Perceived Social Support	0.11 (0.08)	1.11 [0.95, 1.30]	.170
Recovering Quality of Life	-0.01 (0.02)	0.99 [0.96, 1.03]	.753
GP Visit Frequency (Past 12 Weeks)	-0.20 (0.08)	0.82 [0.70, 0.96]	.014
Treatment Credibility	0.10 (0.05)	1.11 [0.99, 1.23]	.063
Treatment Expectancy	0.01 (0.00)	1.01 [1.00, 1.02]	.030
LGBTQA+ Status (ref = non-LGBTQA+)	-0.13 (0.17)	0.88 [0.63, 1.22]	.447
CALD Background (ref = non-CALD)	-0.12 (0.16)	0.89 [0.65, 1.21]	.444

Note. Treatment condition and mini-trial number were included in the model as control variables but are not interpreted here. The intercept is omitted. B = unstandardised logistic regression coefficient (log odds); SE = standard error; OR = odds ratio; CI = confidence interval.

Table S17

Full Model Including Treatment × Baseline Anxiety Severity Interaction (Distress Remission Outcome)

Predictor	B (SE)	OR [95% CI]	p-value
Treatment: Sleep Hygiene	0.24 (0.61)	1.27 [0.39, 4.17]	.692
Treatment: Mindfulness	-0.76 (0.64)	0.47 [0.13, 1.63]	.232
Treatment: Physical Activity	-0.18 (0.61)	0.83 [0.25, 2.73]	.762
Baseline Anxiety Severity	-0.10 (0.05)	0.90 [0.81, 1.00]	.059
Mini-trial Number	0.06 (0.02)	1.06 [1.01, 1.11]	.021
Sex (ref = female)	0.04 (0.20)	1.04 [0.70, 1.55]	.839
Socioeconomic Status	-0.03 (0.05)	0.97 [0.88, 1.07]	.556
Baseline Depression Severity	-0.09 (0.02)	0.92 [0.89, 0.95]	<.001
Perceived Social Support	-0.07 (0.08)	0.93 [0.79, 1.09]	.374
Recovering Quality of Life	0.04 (0.02)	1.04 [1.00, 1.08]	.066
GP Visit Frequency (Past 12 Weeks)	-0.18 (0.10)	0.84 [0.69, 1.01]	.059
Treatment Credibility	0.09 (0.06)	1.10 [0.98, 1.23]	.098
Treatment Expectancy	0.01 (0.00)	1.01 [1.00, 1.02]	.204
LGBTQA+ Status (ref = non-LGBTQA+)	-0.06 (0.18)	0.94 [0.67, 1.33]	.743
CALD Background (ref = non-CALD)	0.05 (0.16)	1.05 [0.76, 1.45]	.778
Sleep Hygiene × Baseline Anxiety	-0.02 (0.06)	0.98 [0.87, 1.11]	.777
Mindfulness × Baseline Anxiety	0.07 (0.06)	1.08 [0.96, 1.21]	.213
Physical Activity × Baseline Anxiety	0.05 (0.06)	1.05 [0.93, 1.18]	.430

Note. B = unstandardised logistic regression coefficient (log odds); SE = standard error; OR = odds ratio; CI = confidence interval.

Table S18

Full Model Including Treatment × Baseline Anxiety Severity Interaction (Distress Response Outcome)

Predictor	B (SE)	OR [95% CI]	p-value
Treatment: Sleep Hygiene	1.58 (0.75)	4.86 [1.12, 21.15]	.035
Treatment: Mindfulness	0.54 (0.77)	1.72 [0.38, 7.77]	.480
Treatment: Physical Activity	0.98 (0.75)	2.66 [0.61, 11.66]	.195
Baseline Anxiety Severity	0.04 (0.04)	1.04 [0.96, 1.13]	.308
Mini-trial Number	0.06 (0.02)	1.06 [1.02, 1.11]	.009
Sex (ref = female)	0.07 (0.19)	1.07 [0.73, 1.56]	.730
Socioeconomic Status	-0.05 (0.05)	0.96 [0.87, 1.05]	.329
Baseline Depression Severity	-0.02 (0.01)	0.98 [0.96, 1.00]	.067
Perceived Social Support	0.11 (0.08)	1.12 [0.96, 1.31]	.148
Recovering Quality of Life	-0.01 (0.02)	0.99 [0.96, 1.03]	.708
GP Visit Frequency (Past 12 Weeks)	-0.21 (0.08)	0.81 [0.69, 0.95]	.012
Treatment Credibility	0.10 (0.05)	1.11 [1.00, 1.23]	.056
Treatment Expectancy	0.01 (0.00)	1.01 [1.00, 1.02]	.029
LGBTQA+ Status (ref = non-LGBTQA+)	-0.13 (0.17)	0.88 [0.63, 1.23]	.443
CALD Background (ref = non-CALD)	-0.11 (0.16)	0.90 [0.66, 1.22]	.486
Sleep Hygiene × Baseline Anxiety	-0.10 (0.05)	0.91 [0.83, 0.99]	.038
Mindfulness × Baseline Anxiety	0.00 (0.04)	1.00 [0.91, 1.08]	.921
Physical Activity × Baseline Anxiety	-0.01 (0.04)	0.99 [0.91, 1.08]	.811

Note. B = unstandardised logistic regression coefficient (log odds); SE = standard error; OR = odds ratio; CI = confidence interval.
